# Are there subgroup differences in the accuracy of ‘screening’ questions for mood and anxiety disorder diagnostic interviews?

**DOI:** 10.1002/mpr.70008

**Published:** 2024-11-07

**Authors:** Matthew Sunderland, Tim Slade

**Affiliations:** ^1^ The Matilda Centre for Research in Mental Health and Substance Use University of Sydney Sydney New South Wales Australia

**Keywords:** anxiety, differential item functioning, measurement bias, mood, population‐based surveys, screening

## Abstract

**Objective:**

To examine the impact of potential measurement bias (i.e., differential item functioning [DIF]) across sex, age, employment, location, and substance use disorders on the screening properties of epidemiological surveys that utilise screening questions when estimating prevalence of mood and anxiety disorders.

**Methods:**

Data comprised of 15,893 respondents who completed the 2020–2022 Australian National Survey of Mental Health and Wellbeing. Questions from the screening module of the Composite International Diagnostic Interview 3.0 were analysed using confirmatory factor analysis and DIF across subgroups of interest. Sensitivity, specificity, and classification rate were derived and compared across models that did and did not adjust for significant levels of DIF.

**Results:**

Sources of DIF were identified across the items was due to age and sex at birth with relatively fewer items displaying DIF across employment, location, and substance use disorders. In terms of screening, the absolute differences in sensitivity and specificity between the DIF‐free and DIF models ranged from 0.001 to 0.091.

**Conclusions:**

The current study found some evidence of DIF in the screening questions used to evaluate mental health disorder prevalence. However, the overall influence of DIF on screening into at least one mood and anxiety disorder module was found to be minimal.

## 1 INTRODUCTION

To derive psychiatric epidemiological information, population‐based surveys predominantly utilise lay interviewer‐administered interviews, such as the World Mental Health Composite International Diagnostic Interview (WMH‐CIDI), the Alcohol Use Disorder and Associated Disabilities Interview Schedule‐5 (AUDADIS‐5), or the Mini International Neuropsychiatric Interview (MINI) (Grant et al., 2015; Kessler & Üstün, 2004; Sheehan et al., 1998). A primary feature of these interviews is the inclusion of a screening section composed of several questions (also called gate questions, skip logic, conditional branching, etc.) that partially tailor the interview to each respondent, reduce the overall interview length, and therefore reduce respondent burden. These screening questions are intended to assess the core symptoms of mood and anxiety disorders and determine whether respondents should proceed to the full diagnostic module. The comprehensive diagnostic criteria are then determined using extensive questioning in the full module. However, if respondents do not meet the initial screening criteria, the full set of diagnostic questions is not administered, and a negative diagnosis for each mood or anxiety disorder is assumed and assigned (Scorza et al., 2015).

This screening approach rests on the assumption of measurement invariance, meaning that individuals from various sociodemographic backgrounds interpret, understand, and respond to the screening questions in the same manner (Leitgöb et al., 2023). Group comparisons in prevalence estimates hinge on this assumption. However, if responses screening questions are influenced by factors other than the actual experience of core symptoms, this reflects measurement non‐invariance. In such cases, the differences in responses may be due to biases specifically related to group membership rather than genuine differences in symptom experience, complicating the validity of group comparisons. For example, females have typically demonstrated increased rates of mood and anxiety disorders in comparison to males, some explanations for the difference include the potential for social‐related response bias where proportion of male respondents see it as socially undesirable to report mood or anxiety symptoms, therefore resulting in a pattern of measurement non‐invariance (Berger et al., 2012; Cavanagh et al., 2016).

Fortunately, it is possible to examine and test the assumption of measurement invariance for the CIDI gate questions through Item Response Theory (IRT), specifically by using tests of differential item functioning (DIF) (Edelen et al., 2006; Teresi & Fleishman, 2007). Numerous studies have applied DIF analyses across various measures, diagnoses, and individual indicators in psychiatric epidemiological surveys to determine measurement invariance (Teresi et al., 2008). If assumption of measurement invariance is not justified, the recommendation is to examine the overall substantive impact of DIF on prevalence or to revise the survey content to better suit subgroups where DIF is present. Indeed, the importance of detecting DIF in epidemiological research has been previously highlighted in a review by Jones (2019). Despite this, there is limited evidence regarding the measurement invariance of the CIDI gate questions across multiple sociodemographic and clinical groups. One notable exception explored age‐related DIF in the CIDI gate items for mood and anxiety disorders within the Australian population, as well as using a different instrument in a US‐based survey, concluding that although minor levels of DIF were present, their impact on population level differences across age groups was minimal (Buchan et al., 2014).

Interpreting the substantive influence of items that exhibit DIF is challenging, particularly regarding how they affect the overall screening ability of the item set to direct respondents into mood or anxiety modules or not. A recent approach, as outlined by Gonzalez and Pelham (2021), quantifies the differences in screening characteristics (e.g., sensitivity, specificity, overall percent agreement) between groups when DIF is considered and when it is not. This approach can determine whether the screening properties are significantly influenced by ignoring potential DIF across groups and therefore would result in problematic comparisons of prevalence that hinge on the accuracy of the gate questions. On the other hand, if the screening properties remain robust despite potential DIF, this would support the continued use of the gate items for comparing prevalence of mood and anxiety disorders across key subgroups.

To date, no study has examined the impact of DIF on the screening properties of epidemiological surveys that utilise screening questions for common mood and anxiety disorders across multiple sociodemographic groups. The current study aims to fill this gap by (1) using data from a recent large population‐based survey to investigate the presence or absence of DIF on the CIDI screening questions, and (2) determine the impact of DIF on the screening properties across key characteristics such as sex, age, employment status, living location, and substance use disorders.

## 2 METHOD

### 2.1 Sample

The current study used data from the 2020–2022 National Survey of Mental Health and Wellbeing (NSMHWB), a national study of the Australian population funded by the Australian Government Department of Health and Aged Care and administered by the Australian Bureau of Statistics (ABS) under the authority of the Census and Statistics Act 1905. The sample recruited within scope Australian households, excluding very remote parts of Australia as well as discrete Aboriginal and Torres Strait Islander communities. Using a stratified area sampling strategy, private households were randomly selected with one usual resident aged 16–85 randomly selected to complete the questionnaire. People aged 16–24 years were oversampled (e.g., had a higher probability of select) to improve estimates for this age group. The 2020–2022 NSMHWB was carried out across two separate cohorts, the first conducted in December 2020 to July 2021 and the second between December 2021 and October 2022. Both cohorts were combined into a single cohort and analysed in the current study. In total, there were 15,893 fully responding household that represent a response rate of 52%. Further methodological and technical details for the survey are provided elsewhere (Australian Bureau of Statistics, 2020–2022).

### 2.2 Measures

Additional modules were included in the NSMHWB to capture sociodemographic and other relevant information, including sex at birth (male, female), age (younger = 16–44 years of age, older = 45–85 years of age), employment status (employed/not in the labour force, unemployed), and location (inner and outer regional/remote and very remote, metro). Location was captured using the ABS remoteness area codes based on the Australian Statistical Geography Standard designed to identify major cities, inner regional, outer regional, remote, and very remote parts of Australia (Australian Bureau of Statistics, 2021–2026). For the current study respondents residing in the major cities were compared against respondents from a composite of inner and outer regional and remote and very remote areas.

The primary diagnostic instrument was the WMH‐CIDI (version 3.0), that examined disorders based on both the Diagnostic and Statistical Manual of Mental Disorders (4th edition) and the International Classification of Diseases (10th edition). The WMH‐CIDI is a lay interviewer‐administered computer‐assisted interview that has been used extensively as part of the global World Mental Health Survey Initiative and has previously demonstrated high levels of concordance with a fully structured clinical diagnostic interview (Kessler et al., 2004). The WMH‐CIDI obtains information over the lifetime on the presence or absence of mood (depressive disorder, dysthymia, and bipolar affective disorder), anxiety (agoraphobia, panic disorder, social anxiety disorder, generalised anxiety disorder, obsessive‐compulsive disorder, and post‐traumatic stress disorder), and substance use disorders (including alcohol, cannabis, sedative, stimulant, and opioid use disorders). The current study focuses on the screening module of the WMH‐CIDI that administers gate questions for depressive disorder, dysthymia, bipolar affective disorder, panic disorder, agoraphobia, generalised anxiety disorder, and social anxiety disorder. A series of 19 questions is administered and collapsed into 9 screening rules (see Table 1) that dictate whether subsequent modules are administered or skipped. For the current study the 9 screening rules were coded dichotomously as either ‘screen in’ or ‘screen out’ for each respondent. Finally, a composite substance use disorder variable was created to examine DIF in the internalising screening items across those with and without any substance use disorder (comprising alcohol use, cannabis use, sedative use, stimulant use, and opioid use disorders).

**TABLE 1:** Wording of the gate questions and screening rules for the internalising modules.

Name	Item/s	Screening rule
SC20	SC20. Have you ever in your life had an attack of fear or panic when all of a sudden you felt very frightened, anxious, or uneasy?	If SC20 = Yes OR SC20a = Yes then Screen IN
	SC20a. Have you ever had an attack when all of a sudden you became very uncomfortable, you either became short of breath, dizzy, nauseous, or your heart pounded, or you thought you might lose control, die, or go crazy?	Else if SC20 = No/Don't know/Refuse AND SC20a = No/Don't know/Refuse then Screen OUT
SC21	SC21. Have you ever in your life had a period lasting several days or longer when most of the day you felt sad, empty, or depressed?	If SC21 = Yes then Screen IN
		Else if SC21 = No/Don't know/Refused then Screen OUT
SC22	SC22. Have you ever had a period lasting several days or longer when most of the day you were very discouraged about how things were going in your life?	If SC22 = Yes then Screen IN
		Else if SC22 = No/Don't know/Refused then Screen OUT
SC23	SC23. Have you ever had a period lasting several days or longer when you lost interest in most things you usually enjoy like work, hobbies, and personal relationships?	If SC23 = Yes then Screen IN
		Else if SC23 = No/Don't know/Refused then Screen OUT
SC24	SC24. Some people have periods lasting 4 days or longer when they feel much more excited and full of energy than usual. Their minds go too fast. They talk a lot. They are very restless or unable to sit still and they sometimes do things that are unusual for them, such as driving too fast or spending too much money. Have you ever had a period like this lasting several days or longer?	If SC24 = Yes then Screen IN
		Else if SC24 = No/Don't know/Refused then Screen OUT
SC25	SC25. Have you ever had a period lasting 4 days or longer when most of the time you were very irritable, grumpy, or in a bad mood?	If SC25 = Yes AND SC25a = Yes then Screen IN
	SC25a. Have you ever had a period lasting 4 days or longer when most of the time you were so irritable that you either started arguments, shouted at people, or hit people?	Else if SC25 = No/Don't know/Refused OR SC25a = No/Don't know/Refused then Screen OUT
SC26	SC26. Did you ever have a time in your life when you were a ‘worrier’—that is, when you worried a lot more about things than other people with the same problems as you?	IF SC26 = Yes OR SC26a = YES OR SC26b = YES then Screen IN
	SC26a. Did you ever have a time in your life when you were much more nervous or anxious than most other people with the same problems as you?	Else if SC26 = No/Don't know/Refused OR SC26a = No/Don't know/Refused OR SC26b = No/Don't know/Refused then Screen OUT
	SC26b. Did you ever have a period of time lasting 1 month or longer when you were anxious and worried most days?	
SC29	SC29. Looking at page 29 of your booklet, was there ever a time in your life when you felt very afraid or really, really shy with people, like meeting new people, going to parties, going on a date, or using a public bathroom?	If (SC29 = Yes OR SC29a = Yes) AND ((SC29.1 = Yes OR SC29.2 = Yes) AND SC29.3 = Yes) then Screen IN
	SC29a. Was there ever a time in your life when you felt very afraid or uncomfortable when you had to do something in front of a group of people, like giving a speech or speaking in class?	
	SC29.1. Was there ever a time in your life when you became very upset or nervous [SC_WS2: Whenever you were in a social situation/when you had to do something in front of a group]?	
	SC29.2. Did you ever stay away from [SC_WS3: Social situations/situations where you had to do something in front of a group] whenever you could because of your fear?	Else if (SC29 = No/Don't know/Refused AND SC29a = No/Don't know/Refuse) OR ((SC29.1 = No/Don't know/Refuse AND SC29.2 = No/Don't know/Refuse) OR SC29.3 = No/Don't know/Refuse) then Screen OUT
	SC29.3. Do you think your fear was ever much stronger than it should have been?	
SC30	SC30. Looking at page 30 in your booklet, was there ever a time in your life when you felt afraid of either being in crowds, going to public places, travelling by yourself, or travelling away from home?	If SC30 = Yes AND ((SC30.1 = Yes OR SC30.2 = Yes) AND SC30.3 = Yes) then Screen IN
	SC30.1. Was there ever a time in your life when you became very upset or nervous whenever you were in crowds, public places, or travelling?	
	SC30.2. Did you ever stay away from these situations whenever you could because of your fear?	Else if SC30 = No/Don't know/Refused OR ((SC30.1 = No/Don't know/Refuse AND SC30.2 = No/Don't know/Refuse) OR SC30.3 = No/Don't know/Refuse) then Screen OUT
	SC30.3. Do you think your fear was ever much stronger than it should have been?	

*Note*: Screening into at least one of the internalising models requires meeting the criteria for one or more of the above screening rules.

### 2.3 Statistical analysis

The key assumptions of IRT and DIF were first examined via the use of confirmatory factor analysis and tests of global model fit and item fit. A one‐factor confirmatory factor model was fit to the 9 binary indicators in the total sample to generate the factor loadings, communality (h2) values, and global fit statistics (root mean square error of approximation [RMSEA], standardised root mean square residual [SRMSR], comparative fit index [CFI], and the Tucker‐Lewis fit index [TLI]). Good model fit was determined based on traditional cut points with RMSEA and SRMSR ≤0.05 and the CFI and TLI ≥0.95 (Hu & Bentler, 1999) as well as an approach to generate dynamic fit index cut‐offs based on the model specification and sample size of the current study (McNeish & Wolf, 2023). Individual item fit was assessed using item RMSEA values with values ≤0.05 indexing good item fit. The two‐parameter logistic item response model was then fit to the data to generate discrimination (a) and difficulty (b) parameters and item traceline plots (or item characteristic curves) separately for the 9 binary indicators.

Tests of DIF then proceeded using an iterative algorithm to flag items as either anchors or potentially exhibiting significant levels of DIF. The first step in this procedure required the generation of a ‘purified’ anchor set for subsequent formal DIF testing based on a method outlined by Woods (2009). This was established using separate multigroup models testing for differences across the target subgroups in the discrimination and difficulty parameters for each item holding all other items as the temporary DIF‐free anchor sets. Items that demonstrated DIF at this stage were subsequently treated as potential DIF items, whereas other items that demonstrated no DIF were subsequently treated as the purified DIF‐free anchor set in a single model. If all items demonstrated potential DIF in the first stage, the items were ranked and the bottom three items with the smallest differences according to model fit comparisons (e.g., Bayesian Information Criterion values) were subsequently treated as the purified DIF‐free anchor set. The formal DIF testing with the purified DIF‐free anchor set was conducted using overall log‐likelihood ratio tests in a single model with Benjamini‐Hochberg false discovery rate adjusted *p*‐values (Benjamini & Hochberg, 1995).

The impact of significant levels of DIF on the probability of screening into at least one of the CIDI mood or anxiety disorder modules was evaluated using the method outlined by Gonzalez and Pelham (2021) and Millsap and Kowk (2004). Briefly, this method involves estimating factor scores based on two multigroup IRT models, the first model treats all item parameters as equal across the groups of interest (i.e., DIF‐free model) whereas the second model freely estimates the item parameters between groups for items that exhibit DIF, as identified in the formal DIF analysis. The factor score (i.e. location on the underlying latent trait) associated with a pre‐defined cut‐point on the original sum scale is then identified and used to generate classification and agreement statistics across the DIF‐free and DIF models. In the case of the current study, the cut score on the original sum score was set to one or more given that each individual needs to meet the criteria for at least one of the gate rules to screen into a module for mood and anxiety disorders. The sensitivity, specificity, and classification rate are generated by examining those who meet the cut‐point associated with the latent trait score in comparison to those who meet the cut‐point on the original sum score. For example, sensitivity is interpreted as the number of respondents above the cut score on the latent variable whose expected observed score under the DIF model is above the observed cut score. Specificity is interpreted as the number of respondents below the cut score on the latent variable whose expected observed score under the DIF model is below the observed cut score (Gonzalez & Pelham, 2021). These values are generated for each group of interest separately and across the two models. If the level of DIF substantially impacts the cut score in terms of identifying similar respondents who pass through the gate questions, then the DIF model will provide different levels of sensitivity and specificity in comparison to the model that is assumed to be DIF‐free.

## 3 RESULTS

### 3.1 Factor analysis and item response model

**FIGURE 1:**
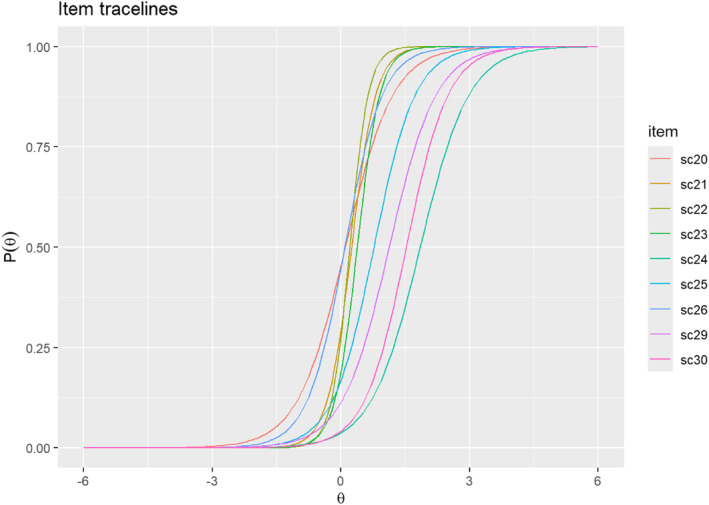
IRT characteristic curves for each screening rule in the total sample. Item definitions are provided in Table 1. IRT, Item Response Theory.

The percent who met the criteria, factor loadings, and IRT item parameters are provided in Table 2 along with the overall model fit of the one‐factor CFA. The results suggest that the one‐factor CFA provided excellent fit to the data with CFI and TLI values >0.95. However, RMSEA and SRMR values were slightly above traditional cut‐offs for excellent fit but were within cut‐offs for good/acceptable fit. The values provided for the dynamic fit index indicate the degree of misspecification potentially impacting the model. The CFI and RMSEA values for the current model indicate that the model does not fit exactly, and the amount of misfit is consistent with somewhere between 2/3 of items with residual correlations greater than 0.3 and all items with residual correlations greater than 0.3 omitted from the model. The individual item fit RMSEA values were all well within the cut‐off for excellent fit suggesting that the IRT model can accurately replicate the observed response rates for each of the individual items in the data. Similarly, standardised factor loadings and communality (h2) values are all >0.3 suggesting high proportions of variance are shared across all items. The item characteristic curves for each of the screening rules are presented in Figure 1, indicating that excitement/full of energy (SC24), social anxiety (SC29), and agoraphobia (SC30) required higher scores on the latent trait to screen positive whereas panic (SC20) and generalised anxiety disorder (SC26) required relatively lower scores on the latent trait. The two most discriminating screening rules were anhedonia (SC22) and discouragement/low life satisfaction (SC23).

**TABLE 2:** Item response characteristics and factor loadings of the screening rules in the total sample.

Rule	%	a	b	*λ*	h2	RMSEA
SC20	47.2	1.791	0.102	0.725	0.525	0.012
SC21	41.5	3.518	0.248	0.900	0.810	0.034
SC22	42.8	4.641	0.206	0.939	0.881	0.036
SC23	36.7	3.799	0.381	0.913	0.833	0.028
SC24	9.5	1.752	1.849	0.717	0.514	0.023
SC25	27.8	2.069	0.770	0.772	0.596	0.022
SC26	42.3	2.280	0.092	0.801	0.642	0.014
SC29	20.7	1.822	1.126	0.731	0.534	0.021
SC30	12.3	2.048	1.526	0.769	0.591	0.020
Model fit						
RMSEA = 0.078	SRMSR = 0.051	CFI = 0.971	TLI = 0.962			

*Note*: Item definitions are provided in Table 1.

Abbreviations: a, IRT discrimination parameter; b, IRT difficulty parameter; CFI, comparative fit index; h2, communality; RMSEA, root mean square error of approximation; SRMSR, standardised root mean square residual; TLI, Tucker‐Lewis fit index; *λ*, factor loading.

### 3.2 Differential item functioning

The results of the final DIF testing after identification of the purified DIF‐free anchor set for each of the sociodemographic variables of interest are provided in Table 3. Several items demonstrated significant levels of DIF associated with age and sex at birth whereas relatively fewer items displayed significant DIF across substance use disorders, employment, and location. For screening items that targeted panic disorder, major depression, agoraphobia, and generalised anxiety disorder, males exhibited increased difficulty (b), suggesting that male respondents tend to have a decreased probability of screening into these modules compared to female respondents, despite holding latent scores constant. These items also demonstrated reduced discrimination (a) in males and therefore tended to be poorer indicators of the underlying latent trait in comparison to females. Similarly, for older respondents the difficulty parameters for screening items that target irritability, worry, and social anxiety were increased, suggesting older respondents have a decreased probability of screening into these modules compared to younger respondents, despite holding latent scores constant. However, the reverse was true for older adults in terms of screening into modules for depression compared to younger respondents. In terms of the presence of any substance use disorder, the difficult parameters were lower for screening into modules targeting anhedonia and excitement/full of energy and higher for screening into the depression module in comparison to people who do not have any substance use disorder.

**TABLE 3:** Item response parameters from tests of differential item functioning across groups.

	Sex at birth				Age				Employment status				Location				Substance use			
	Female (ref)		Male		Younger (ref)		Older		Employed/NILF (ref)		Unemployed		Regional/Remote (ref)		Metro		No (ref)		Yes	
	a	b	a	b	a	b	a	b	a	b	a	b	a	b	a	b	a	b	a	b
SC20	1.844	−0.084	1.561	0.102	1.856	−0.041	‐	‐	1.783	0.111	‐	‐	1.781	0.106	‐	‐	1.771	0.130	‐	‐
SC21	3.382	0.145	‐	‐	3.869	0.154	3.657	0.054	3.550	0.254	2.407	0.421	3.498	0.252	‐	‐	3.495	0.273	3.533	0.420
SC22	4.980	0.128	3.965	0.071	4.847	0.057	‐	‐	4.620	0.215	‐	‐	4.615	0.210	‐	‐	4.575	0.234	‐	‐
SC23	3.948	0.308	3.346	0.257	3.941	0.227	‐	‐	3.781	0.391	‐	‐	3.778	0.386	‐	‐	3.804	0.410	2.573	0.394
SC24	1.866	1.827	1.571	1.745	1.582	1.665	2.020	1.563	1.745	1.867	‐	‐	1.742	1.863	‐	‐	1.730	1.914	1.371	1.779
SC25	1.981	0.690	‐	‐	2.175	0.479	2.047	0.735	2.057	0.783	‐	‐	2.057	0.777	‐	‐	2.047	0.805	‐	‐
SC26	2.285	−0.060	2.051	0.04	2.327	−0.124	2.360	0.003	2.272	0.101	‐	‐	2.266	0.096	‐	‐	2.254	0.120	‐	‐
SC29	1.743	1.061	‐	‐	1.809	0.850	1.887	1.060	1.815	1.140	‐	‐	1.881	1.198	1.793	1.116	1.801	1.166	‐	‐
SC30	1.996	1.386	1.899	1.627	2.102	1.341	‐	‐	2.037	1.543	‐	‐	2.036	1.538	‐	‐	2.020	1.573	‐	‐

*Note*: Missing cells in the focal groups represent items that do not display DIF and their item parameters are fixed to the reference group. Item definitions are provided in Table 1.

Abbreviations: a, IRT discrimination parameter; b, IRT location parameter; NILF, not in the labour force.

### 3.3 Impact of DIF on screening

The overall screening impact (sensitivity, specificity, classification) depending on whether item level DIF across sociodemographic groups is ignored or accounted for in the model is provided in Table 4. The results suggest that despite the evidence of statistically significant levels of DIF across multiple items, the impact on the screening properties of the mood or anxiety screening questions across different socio‐demographic groups is minimal. The absolute differences in sensitivity and specificity between the DIF‐free and DIF models ranged from 0.001 to 0.091. The largest difference occurred in the specificity between people who did and did not have any substance use disorder. The model accounting for DIF led to a lower likelihood of the gate questions accurately identifying respondents who *should not screen* into the mood or anxiety modules for those who with a substance use disorder compared to those who do not.

**TABLE 4:** Sensitivity and specificity of the screening cut‐point in models ignoring and account for DIF across subgroups of interest.

	Ignoring DIF		Accounting for DIF		Difference^a^	
	Female	Male	Female	Male	Female	Male
Sens	0.914	0.906	0.915	0.907	−0.001	−0.001
Spec	0.757	0.787	0.762	0.780	−0.005	0.007
Classification rate	0.877	0.867	0.878	0.865	−0.001	0.002
	Younger	Older	Younger	Older	Younger	Older
Sens	0.917	0.899	0.924	0.893	−0.007	0.006
Spec	0.758	0.777	0.733	0.782	0.025	−0.005
Classification rate	0.879	0.860	0.878	0.858	0.001	0.002
	Employed	Unemployed	Employed	Unemployed	Employed	Unemployed
Sens	0.906	0.942	0.908	0.944	−0.002	−0.002
Spec	0.776	0.705	0.772	0.682	0.004	0.023
Classification rate	0.896	0.900	0.870	0.898	0.026	0.002
	Regional/Remote	Metro	Regional/Remote	Metro	Regional/Remote	Metro
Sens	0.911	0.915	0.906	0.912	0.005	0.003
Spec	0.750	0.762	0.762	0.768	−0.012	−0.006
Classification rate	0.866	0.873	0.866	0.872	0.000	0.001
	No substance	Substance	No substance	Substance	No substance	Substance
Sens	0.905	0.971	0.906	0.977	−0.001	−0.006
Spec	0.766	0.773	0.765	0.682	0.001	0.091
Classification rate	0.865	0.965	0.865	0.968	0.000	−0.003

Abbreviations: DIF, differential item functioning; Sens, sensitivity; spec, specificity.

^a^ Represents difference between sensitivity, specificity, and classification rate in the model that ignores DIF versus model that accounts for DIF.

## 4 DISCUSSION

The study sought to examine the presence of DIF in the gate questions that determine who screens in or out of the diagnostic modules of common mood and anxiety disorders in the WMH‐CIDI, a measure that has been used regularly in large scale epidemiological studies across the globe. Moreover, the study examined the potential impact of DIF on the screening properties of the WMH‐CIDI gate questions. The main source of DIF identified across the items was due to age and sex at birth. Of the nine items, six demonstrated significant levels of DIF across sex at birth, five demonstrated significant DIF across age, three items across people with and without any substance use disorder, and one item each across location and education. Despite the level of DIF identified across sociodemographic groups, the overall impact on screening into the modules for mood and anxiety disorders was minimal, and potentially ignorable when comparing differences in prevalence across subpopulations. These results provide more confidence in the population‐level differences previously identified in studies that use the WMH‐CIDI, and specifically the screening approach to estimate prevalence of mood and anxiety disorders in general.

The results identified in the current study were broadly consistent with age‐related investigations of DIF conducted previously in three other large‐scale epidemiological surveys conducted in Australia and in the United States of America (Buchan et al., 2014). Importantly, both studies utilised different but related methods to detect DIF, with the current study further extending these findings by specifically focussing on the screening properties of the CIDI items, providing additional robustness to the finding that external factors related to age are not substantially biasing differences in prevalence.

With respect to sex‐related DIF in the items, the findings reflect similar patterns to those found in prior studies that demonstrate sex‐related differences in prevalence. Namely, males tended to underreport anxiety symptoms whereas females tended to underreport items for discouragement, anhedonia, and excitability, despite being matched in terms of the underlying trait. This pattern of sex‐related bias may be explained by persistent and changing social norms in the expression and reporting of mood or anxiety symptoms (Cavanagh et al., 2016). Females have traditionally reported increased mood and anxiety symptoms in comparison to males in general. However, the current study would imply that males continue to under‐report anxiety, worry and panic‐like symptoms whereas females are increasingly likely to under‐report affective and bipolar like symptoms. Indeed, it might be that this pattern of individual item responding across the sex groups results in minimal impact on screening overall. For example, similar numbers of males and females may continue to screen into the mood or anxiety disorder modules in general, regardless of the specific modules that they screen into, given the different directions of individual item DIF cancel each other out. As such, more females might screen into anxiety modules and more males might screen into affective‐bipolar modules, despite being matched in terms of underlying liability.

The finding that the screening impact on mood and anxiety disorders in general across groups of interest is not substantially impacted by individual disorder levels of DIF across groups, potentially highlights the value of utilising broad or common latent variables to capture psychopathology in epidemiological studies in the future (Conway et al., 2019; Kotov et al., 2022). The use of latent variables to capture commonalities and co‐occurrence across disorders appears to be more robust against levels of disorder‐specific DIF across groups, whereas DIF in some of the specific gate questions could potentially lead to bias when comparing rates of specific disorders. Whilst the use of broad latent variables does come at a cost of specificity, the added value of this specificity when making clinical and policy decisions is uncertain and requires ongoing investigation. Previously, studies have demonstrated that the use of latent variables that capture ‘internalising’ disorders accounts for the majority of variance when examining associations with a range of salient and clinically relevant factors, such as novel biomarkers, illness course, temperament, personality, and treatment response (Lynch et al., 2021; Watson et al., 2022).

There are some limitations associated with the study that need to be considered when interpreting the findings. First, not all subgroups with potentially different sociodemographic were considered in scope for the NSMHWB, for example, those who live in remote parts of Australia, those who live in discrete Aboriginal and Torres Strait Islander communities, people who are homeless, people in prison, or living in nursing homes or other care facilities. Likewise, the response rate of 52% is less than ideal and may introduce some degree of sampling bias in the results. The method used to determine the presence of DIF and impact on screening did not account for the survey weight. Whilst survey weights can be incorporated into the analysis, the use of raw or normalised weights and the impact on latent variable estimates introduces an increased level of complexity. A prior simulation study has shown that applying raw or normalised sample weights yields smaller standard errors and in turn larger test statistics in latent variable models, potentially inflating the probability of detecting significant levels of DIF for the current study, albeit ignoring the sample weights has little effect on the standard errors relative to the standard deviation of the estimates (Kaplan & Ferguson, 1999). Instead, the results implicitly assume that the data were collected using simple random sampling and therefore the unequal probability of selection was ignored and may result in some degree of bias in the parameter estimates. Caution should be taken when attempting to generalise these findings to the broader Australian population but rather interpreted as applicable to community respondents who participated in this large sample. Finally, with respect to the generalisability of the findings, it should be cautioned that these findings may not adequately generalise to the formation of a clinical diagnosis and clinical assessment more broadly. Rather, these findings are interpretable acknowledging various implicit assumptions to diagnostic assessment made by lay‐administered structured diagnostic interviews and their implementation in epidemiological studies. Additional clinical reappraisal studies might result in different conclusions.

The current study found some evidence of DIF in the gating questions and screening modules of the WMH‐CIDI that is used to determine mental health disorder prevalence across numerous countries around the globe. The evidence of DIF primarily emerge across sex and age with some evidence of DIF in a smaller number of items across substance use, location, and employment status. Despite potentially differential patterns of screening into the mood and anxiety disorder modules across these groups of interest, the overall influence of screening into at least one mood and anxiety disorder module (regardless of the specific disorder) was found to be minimal and would potentially not influence observed comparisons between subgroups within the sample when utilising broader indicators of psychopathology.

## AUTHOR CONTRIBUTIONS

**Matthew Sunderland**: Conceptualization; methodology; software; formal analysis; visualization; writing—review & editing; writing—original draft. **Tim Slade**: Conceptualization; methodology; supervision; writing—review & editing.

## CONFLICT OF INTEREST STATEMENT

The authors report no conflict of interest.

## Data Availability

The data that support the findings of this study are available from the Australian Bureau of Statistics. Restrictions apply to the availability of these data, which were used under licence for this study. Data are available from https://www.abs.gov.au/ with the permission of the Australian Bureau of Statistics.
